# Supplementary figures and images for: Mevalonate Pathway-mediated ER Homeostasis Is Required for Haploid Stability in Human Somatic Cells
Source: Cell Struct Funct. 2020 Dec 22;46(1):1–9. doi: 10.1247/csf.20055 (PMC10511059; doi:10.1247/csf.20055)

Figure S1

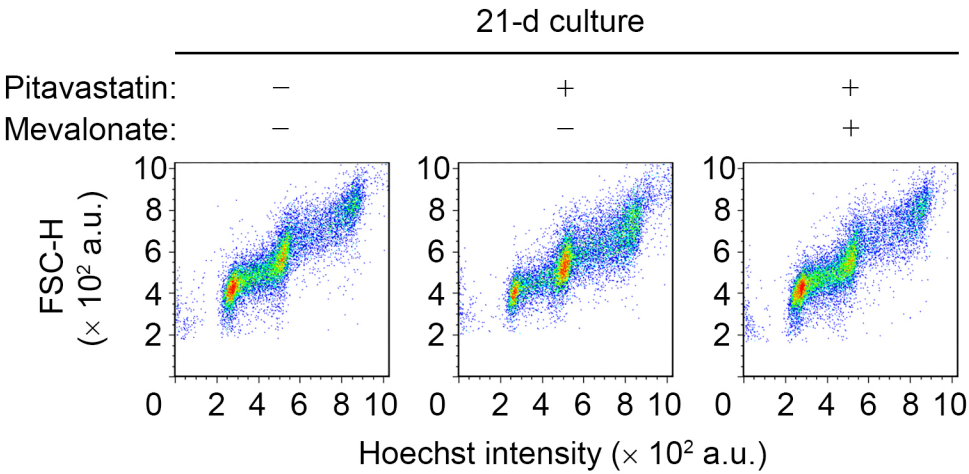

Supplement: Supplementary file 1 — Fig. S1 [file csf_46_20055_1.pdf]
