# Supplementary material for: Mevalonate Pathway-mediated ER Homeostasis Is Required for Haploid Stability in Human Somatic Cells
Source: Cell Struct Funct. 2020 Dec 22;46(1):1–9. doi: 10.1247/csf.20055 (PMC10511059; doi:10.1247/csf.20055)
Supplement: Supplementary file 2 — Table S1 [file csf_46_20055_2.pdf]

Table S1: A list of antibodies used in this study

**Antibody**	**Species**	**Dilution**	**Company (Catalog#)**
ATF6	Mouse	1:500	Santa Cruz (sc-166659)
IRE1α	Rabbit	1:1000	Cell signaling technology (14C10)
PERK	Rabbit	1:1000	Cell signaling technology (C33E10)
ATF4	Rabbit	1:1000	Cell signaling technology (D48B)
CHOP	Mouse	1:1000	Cell signaling technology (L63F7)
β-tubulin	Mouse	1:1000	Wako (10G10)
Horseradish peroxidase-conjugated anti-Mouse IgG	Goat	1:1000	Jackson immune research (115-035-003)
Horseradish peroxidase-conjugated anti-Rabbit IgG	Goat	1:1000	Jackson immune research (111-035-003)
