# Supplementary material for: Mevalonate Pathway-mediated ER Homeostasis Is Required for Haploid Stability in Human Somatic Cells
Source: Cell Struct Funct. 2020 Dec 22;46(1):1–9. doi: 10.1247/csf.20055 (PMC10511059; doi:10.1247/csf.20055)
Supplement: Supplementary file 3 — Supplemental materials and methods [file csf_46_20055_3.pdf]

Mevalonate pathway-mediated ER homeostasis is required for haploid stability in human somatic cells

Supplemental materials and methods

Cholesterol measurement

For total cellular cholesterol measurement, cells were once washed with DPBS, resuspended in 850 μL DPBS, and lysed by sonication. Fifty μL cell lysis was mixed with 50 μL 0.1 M NaOH, incubated at 60°C for 2 h, and subjected to total protein measurement using Protein Assay Bicinchoninate kit (06385-00, nacalai tesque). For cholesterol extraction, the remaining cell lysis was mixed with 1 mL chloroform and 2 mL methanol, and incubated at 37°C for 2 h with vigorous agitation. After centrifugation at 1000 × *g* for 5 min, cholesterol extract was collected from the bottom layer. Cholesterol was further extracted from the remaining lysis by repeating the addition of 2 mL chloroform and centrifugation 3 times. The cholesterol extract was evaporated under a stream of N_2 and dissolved in 100 μL DPBS. The extracted total cholesterol was measured using LabAssay Cholesterol kit (294-65801, Wako) according to the manufacture's instruction. The oxidized and condensated N-Ethyl-N-(2-hydroxy-3-sulfopropyl)-3,5-dimethoxyaniline, sodium salt and 4-Aminoantipyrin were measured with 590 nm wavelength absorbance using iMark microplate reader (BIO-RAD). Cholesterol amount is then normalized to total cellular protein amount for comparison. For visualization of intracellular cholesterol, the Cholesterol Cell-Based Detection Assay Kit (10009779, Cayman Chemical) was used according to the manufacture's instruction.
